# Integrated metabolomics and intestinal microbiota analysis to reveal anti-post-weaning diarrhea mechanisms of Modified Yupingfeng Granule in Rex rabbits

**DOI:** 10.3389/fmicb.2025.1470731

**Published:** 2025-04-10

**Authors:** Dongbo Li, Chao Li, Ning Liu, Hanzhong Liu, Zhiju Yu, Quanjin Liu, Gang Shu, Juchun Lin, Wei Zhang, Guangneng Peng, Ling Zhao, Huaqiao Tang, Haohuan Li, Funeng Xu, Hualin Fu

**Affiliations:** ^1^Department of Pharmacy, College of Veterinary Medicine, Sichuan Agricultural University, Chengdu, Sichuan, China; ^2^Sichuan Academy of Grassland Sciences, Chengdu, Sichuan, China

**Keywords:** Modified Yupingfeng Granule, post-weaning diarrhea, intestinal microbiota, metabolomics, Rex rabbits

## Abstract

**Introduction:**

Post-weaning Diarrhea (PWD) is a kind of physiological stress diarrhea in Rex rabbits after weaning, which can lead to death in severe cases. Traditional Chinese medicine (TCM) has been widely used in animal due to its advantages of natural origin, diverse functions, safety, reliability, economy and environmental protection. Modified Yupingfeng Granule (MYPFG) is an improved Yupingfeng prescription based on the famous traditional Chinese prescription Yupingfeng (YPF), which is combined with other TCM and has obvious synergistic and additive activity in order to obtain an excellent natural medicine for PWD.

**Methods:**

In this study, 120 weaned Rex rabbits were randomly allocated to 4 treatment groups, including control (CON), low dose (LD), medium dose (MD), high dose (HD). Rabbits were fed a control diet or a different MYPFG proportions of diet for 30 days. The study combined 16S rRNA analysis of intestinal microbiota and cecal contents metabolomics to explore the MYPFG effect on weaned Rex rabbits.

**Results:**

MYPFG increased average daily gain, villus length to crypt depth ratio and decreased the feed to meat ratio, diarrhea frequency, mortality rate, depth of crypt (*p* < 0.05). The intestinal microbiota test found that MYPFG could change the abundances of Patescibacteria, *Sphingobium*, *Ruminococcus,* and *Oxalobacter*. Metabolomics analysis found that effect may be related to its regulation of Glycine, serine and threonine metabolism, Arginine and proline metabolism. Nicotinate and nicotinamide metabolism.

**Discussion:**

MYPFG could regulate intestinal microbiota and change the metabolic pathway of some amino acids to alleviate the PWD in Rex rabbits.

## 1 Introduction

Rex rabbits (*Oryctolagus Cuniculus*), known for their excellent fur, rapid growth, and delicious and nutritious meat, are valuable animals with high economic potential (Gao-yun, 2017). However, they are prone to diarrhea and even death after weaning, which can significantly reduce their breeding success and affect economic benefits (Zhi-ping, 2017). Changes in the intestinal microbiota may play an important role in the development of diarrhea (Zhao L. et al., 2025). Studies have shown that the intestinal microbiota of rabbits undergoes significant changes at different developmental stages, especially after weaning and the cessation of breast milk (Fu et al., 2024). It has been reported that regulating the intestinal microbiota can improve the function of the intestinal mucosa and that altering the intestinal microbiota can enhance intestinal barrier function and have a positive effect on intestinal tight junction proteins (Soufi et al., 2024; Zhao L. et al., 2025). Intestinal barrier damage in rabbits also affects the regulation of the intestinal microbiota (Fan et al., 2024). When Rex rabbits are weaned, the type of food is changed while the intestinal villi are not yet fully developed, leading to the destruction and invasion of the intestinal mucosa by undigested products and toxins (Hongli et al., 2017). Meanwhile, changes in endogenous metabolites and the intestinal microbiota further exacerbate the symptoms of diarrhea and can lead to death. The intestinal microbiota of newborn rabbits has a higher abundance of lipid-metabolizing bacteria, which helps the host extract more energy from breast milk lipids. It was reported that the abundance of *Clostridium* and *Ruminococcus* significantly increased after weaning (Zhao M. et al., 2024), assisting the rabbit in digesting solid feed. The introduction of solid feed is an important driving factor affecting the composition of the rabbit intestinal microbiota. Solid feed can disturb the intestinal microbiome, potentially leading to heat stress, reduced antioxidant defense, and increased inflammation risk, thereby triggering diarrhea (Lei et al., 2019).

Traditional Chinese medicine (TCM) contains a large number of beneficial substances, such as polysaccharides, flavonoids, and saponins (JiaHua et al., 2022; Zhe et al., 2022; Yu and Li, 2025). The water extracts of TCM have been used for disease control for thousands of years (Zhe et al., 2022). TCM offers unique advantages in the treatment of diarrhea. Shenling Baizhu powder (SLBZP), a TCM prescription renowned for its efficacy, is specifically recognized for its therapeutic effects in managing diarrhea associated with spleen qi deficiency. It has recently been reported to treat diarrhea induced by a lard diet in mice (Qiao et al., 2024). Another TCM, Sishen pill, has been shown to alleviate diarrhea associated with kidney-yang deficiency syndrome (Xie S. et al., 2024). Similar studies have also shown that Sishen pills effectively treat diarrhea associated with kidney-yang deficiency syndrome by regulating the kidney-intestinal bacteria-metabolic pathway (Li X. et al., 2024). Bohe pill is also a TCM that can treat diarrhea induced by a high-fat and high-protein diet by regulating intestinal mucosal bacteria (Huang et al., 2022). It has been reported that Qiweibaizhu powder is an effective TCM prescription for treating diarrhea (Shao et al., 2020). Meanwhile, TCM and the intestinal microbiota share a common characteristic of adopting a holistic view as their theoretical foundation. TCM, as a medical system based on holistic principles, focuses on the overall balance and regulation of the body (Geng et al., 2025). The intestinal microbiota is a part of the mutual relationship between the intestinal environment and living organisms (Wlazło et al., 2025). The intestinal microbiota participates in the metabolism process, and metabolites further interact with the immune and antioxidant systems (JiaHua et al., 2022). The application of these traditional Chinese medicines can promote the growth of beneficial bacteria, improve animal meat quality, inhibit the reproduction of harmful bacteria, and thus regulate the balance of the intestinal microbiota (Yu and Li, 2025). TCM can influence the immune regulation of the body through the intestinal microbiota. It emphasizes tonifying yin and yang, regulating qi and blood, and restoring the balance of yin and yang in the body through the use of medicinal herbs. TCM can affect the body’s metabolic function by regulating the intestinal microbiota. The intestinal microbiota plays an important metabolic role in the body by breaking down substances in food that are difficult to digest and producing beneficial metabolites. Given the fact that most TCMs are administered orally the intestinal microbiota significantly impacts their metabolism.

In recent years, TCM has shown significant advantages in regulating metabolomics and the intestinal microbiota. Based on the metabolomics and intestinal microbiota analysis, the mechanisms through which traditional Chinese medicine impacts certain diseases have become a popular research hotspot. For example, a study explored the mechanism of Suanzaoren decoction in improving insomnia in rats by integrating metabolomics and intestinal microbiota analysis (He et al., 2022). Research on the mechanism of quercetin in the treatment of hyperlipidemia was based on metabolomics and intestinal microbiota analysis (Tongtong et al., 2023). Some studies have examined the spleen-strengthening effect of TCM through metabolomics and intestinal microbiota analysis (Yanling et al., 2022).

Yupingfeng (YPF), a Chinese herbal formula consisting of *Astragalus*, *Atractylodes*, and Radix Sileris, is a traditional Chinese medicine immunomodulator with a history of use spanning over 800 years (Sun et al., 2017). A recent study, which integrated metabolome, lipidome, and intestinal microbiome analysis, revealed the immunomodulation effects of Astragali Radix in healthy human patients (Gui et al., 2024). Another study also confirmed that it possesses a variety of biological activities, including antioxidant, anti-inflammatory, antibacterial, and anti-stress properties (Zhao W. et al., 2025). *Atractylodes* is a perennial herbaceous plant in the family Asteraceae, and its rhizome is often used in the production of medicines. Polysaccharides, the main active components extracted and isolated from *Atractylodes*, show significant pharmacological activities both *in vivo* and *in vitro*, such as immunomodulatory, antioxidant, antidiabetic, and intestinal protective effects (Lin F. et al., 2024). *Atractylodes* polysaccharides ameliorated colitis by regulating the intestinal microbiota and tryptophan metabolism (Zhang et al., 2024). Radix Sileris could inhibit inflammation and control apoptosis by regulating the PI3K/Akt, p53, and NF-κB signaling pathways to treat enteritis (Wang et al., 2023). Prim-O-glucosylcimifugin, the active componetn in Radix Sileris, has effects such as dispelling wind, relieving exterior symptoms, and providing anti-inflammatory and pain-relieving benefits. Previous research indicates that prim-O-glucosylcimifugin can improve intestinal permeability and visceral sensitivity, inhibit inflammatory reactions, and alleviate various symptoms of diarrhea (Li and Hu, 2024). Furthermore, the clinical effectiveness of YPF in treated diarrhea has been well established (Cheng et al., 2014). YPF has been also used in combination with other herbs to treat diarrhea (Jingxue and Jianguo, 2017). It could improve animal growth performance and immune function (Chu et al., 2023). Additionally, researchers have revealed that YPF promotes intestinal microbiota immunity in mice (Dong-qi et al., 2018). Recent research has found that YPF can promote the growth of chickens by regulating the intestinal microbiota (Guan et al., 2024). Zheng et al. (2023) also found that YPF can improve the immune function and intestinal microbiota of chickens.

Chinese herbal formulas primarily embody the therapeutic principles of treatment based on syndrome differentiation and the combination of complementary herbs in TCM. Considering the poor digestion and decreased immunity in weaned Rex rabbits, Modified Yupingfeng Granule (MYPFG) was developed by incorporating *Bupleurum chinense*, hawthorn, and Shenqu into YPF, based on the principles of liver soothing, Qi replenishment, and digestive support. *Bupleurum chinense* is known to relieve exterior conditions and fever, soothe the liver, alleviate depression, and treat stress-induced diarrhea (Yan-hong and Yan-fang, 2017). Saikoside A, an active ingredient in Bupleurum chinense, has significant anti-inflammatory activities (Lin Y. et al., 2024). It can promote intestinal repair and stabilize the function of the intestinal microbiota by regulating the levels of inflammatory cytokines, functional proteins, and enzyme activities (Jia et al., 2023). Recent research has shown that hawthorn can improve growth, feed conversion, digestive enzymes, and the intestinal microbiota (Wang et al., 2024). Shenqu (medicated leaven) has been reported to aid digestion and is also useful in the treatment of gastric diseases (Beihua et al., 2015). In the early stage of this experiment, we used the method described by researchers (Shao et al., 2017). *Bupleurum chinense*, hawthorn, and Shenqu were used to modify MYPFG. According to the efficacy of each traditional Chinese medicine in the prescription, an orthogonal design was used to optimize the prescription of MYPFG, and the optimal prescription of MYPFG was screened out. The clinical effects (diarrhea rate and mortality) of MYPFG were better than those of the original YPF granule.

There is a close relationship between the intestinal microbiota and TCM (Runzhi et al., 2020). YPF has become a research hotspot for regulating the intestinal microbiota, enhancing immunity, improving animal growth performance, and maintaining intestinal health. For example, YPF could promote the growth of chickens by regulating the intestinal microbiota (Guan et al., 2024). It has been reported that YPF can improve the immune function of chicks and regulate the intestinal microbiota (Zheng et al., 2023). YPF also improved intestinal barrier integrity and suppressed intestinal inflammation (Yao et al., 2023). YPF granules could ameliorate cyclophosphamide-induced immune injury by protecting the intestinal barrier (Huang et al., 2025). YPF can improve the growth performance, intestinal tissue development, and immune system of broiler chicks (Xue et al., 2025). Furthermore, YPF can also improve intestinal health by affecting the intestinal barrier, immune system, and microbiota of *Macrobrachium rosenbergii* (Liu M. et al., 2024). In summary, YPF can promote the growth of beneficial bacteria and inhibit the reproduction of harmful bacteria, thereby regulating the balance of the intestinal microbiota. The application of YPF in animals can improve the intestinal environment, enhance the function of the intestinal immune system, and improve the resistance to diseases. YPF can also improve the intestinal environment, regulate the composition and function of the intestinal microbiota, and affect metabolic processes in the body. Using MYPFG, we can optimize the treatment plan for diarrhea and provide personalized adjustments based on the specific conditions of the intestinal microbiota.

In this study, MYPFG demonstrated a protective effect on the intestines of weaned Rex rabbits, and the main mechanisms included promoting intestinal development, repairing the mucosal barrier, and regulating the intestinal microbiota and endogenous metabolites. These effects may be attributed to MYPFG’s regulation of the structure of the intestinal microbiota and its influence on metabolite changes. The research showed the protective effect of MYPFG on weaned Rex rabbits. The study ultimately integrated 16S rRNA and metabolomics analyses to examine the intestinal function, microbial composition, and metabolites in Rex rabbits with post-weaning diarrhea (PWD) that were treated with MYPFG.

## 2 Materials and methods

### 2.1 Chemicals and reagents

Saikosaponin A and prim-O-glucosylcimifugin were purchased from the Shanghai Yuanye Bio-Technology Co., Ltd. (Shanghai, China). p-Dimethylaminobenzaldehyde and vanillin were purchased from Shanghai Macklin Biochemical Co., Ltd. (Shanghai, China). Hematoxylin and eosin Y were obtained from Sigma-Aldric. Alcian Blue 8GX was purchased from Beijing Solarbio Technology Co., Ltd. (Beijing, China). Other reagents were provided by Chengdu Chron Chemicals Co., Ltd.

### 2.2 Animals

Rex rabbits, approximately 40 days old and weighing approximately 1,000 g, were provided by the Rex Rabbit Research Institute of Sichuan Academy of Grassland Sciences. The animals were given a basal diet and tap water at liberty and were maintained in cages under controlled conditions (23 ± 2°C, 12-h light/dark cycle). All experiments and procedures were carried out in accordance with the Regulations of Experimental Animal Administration issued by the State Committee of Science and Technology of China. The composition and nutrient levels of the basal diet are listed in Table 1. All animal procedures were conducted in accordance with the national standard, Laboratory Animal-Requirements of Environment and Housing Facilities (GB14925-2001), and approved by the Sichuan Agricultural University Institutional Animal Care and Use Committee under permit number CSQ-2020303006.

**Table 1:** The composition and nutrient levels of basal diet (air-dry basis) %.

Items	Content	Nutrient levels	Content
Ingredients		Digestible Energy (DE, MJ/kg)	10.21
Corn	20.0	Crude Protein (CP)	16.00
Bran	22.8	Crude Fiber (CF)	14.29
Peanut vine	40.1	EE	3.02
Soybean meal	14.6	Met+Cys	0.46
NaCl	0.5	Lys	0.56
CaHPO_4_	1.0	Ca	0.58
Premix	1.0	P	0.62
Total	100	I (mg/kg)	0.22

The premix provided the following per kilogram of the diet: Lys, 1.5 g; VA, 8000 IU; VD, 31000 IU; VE, 50 mg; Fe, 100 mg; Cu, 50 mg; Mg, 150 mg; Zn, 50 mg; Mn, 30 mg; and Se, 0.1 mg.

Nutrition levels were calculated values.

### 2.3 Preparation of MYPFG

MYPFG was formulated into granules by adding inactive ingredients to the hot water extract of a mixture of six crude herbs. *Bupleurum chinense*, *Astragalus*, *Atractylodes*, hawthorn, Shenqu, and Radix Sileris were mixed in a dosage ratio of 5:4:4:3:3:4 and wetted with distilled water (1:8, w/v) for 2 h. Then, the herbs were boiled at 100°C for 3 h and filtered through a multi-layer gauze, and the filtrate was collected. After the herbs were repeatedly boiled and filtered three times, all filtrates were mixed. To facilitate the preparation of the granules, the filtrate was concentrated to half the weight of the original Chinese herbal medicine. The extracts were granulated by adding inactive ingredients and wetting agents. The concentration of MYPFG was determined such that each gram of the granules contained 1 g of raw plant material. All the above herbs were purchased from Sichuan Qianyuan Traditional Chinese Medicine Slices Co., Ltd.

### 2.4 Determination of the major components using high-performance liquid chromatography (HPLC)

The quality control of traditional Chinese medicine preparations aims to ensure the stability and clinical efficacy of the preparations by detecting the content of active ingredients in the prescription (Zhuang et al., 2024). In this study, Radix Sileris was the traditional Chinese medicine in the original prescription of YPF, while *Bupleurum chinense* was the representative of the modified medicine. We chose the active ingredient prim-O-glucosylcimifugin in Radix Sileris and the active ingredient Saikoside A in *Bupleurum chinense* as the quality control standards for MYPFG.

MYPFG was analyzed using high-performance liquid chromatography (HPLC) with the Corona ultra Charged Aerosol Detector (CAD) on an Agilent HPLC system. Approximately 0.1 g of the MYPFG was weighed and dissolved in 1 mL of a 5% concentrated ammonia methanol solution. The solution was dissolved using ultrasound, filtered through a 0.45-μM filter, washed twice with methanol, evaporated to dryness, and then diluted with methanol. Saikoside A and prim-O-glucosylcimifugin were dissolved in methanol. The complete sample solution was filtered through a 0.22-μM filter. Subsequently, 20 μL supernatant was injected into the HPLC system for analysis. The chromatographic conditions were as follows: a reverse-phase column (Intersil ODS-3.5 μm, 4.6 mm × 250 mm I.D.) connected to a guard column (C18, 5 μm, 4.6 mm × 10 mm I.D.). The elution flow rate was 1.0 mL/min with a mobile phase gradient of A-B (A: H_2_O; B: acetonitrile), which was varied as follows: 0 ~ 25 min, 97 ~ 5% A and 3 ~ 95% B. The injection volume was 20 μL, and the UV detection wavelengths were set at 210 nm for Saikoside A and 250 nm for prim-O-glucosylcimifugin. The quantitative test results showed that the concentrations of Saikoside A and prim-O-glucosylcimifugin were 49.703 and 18.797 μg/g in the MYPFG, respectively.

### 2.5 Experimental design

A total of 120 Rex rabbits, after 7 days of adaptive feeding, were randomly divided into four groups, with 30 rabbits in each group: control (CON), low dose (LD), medium dose (MD), and high dose (HD). The CON group was fed the basic diet, as shown in Table 1. The MYPFG formulations (0.5, 1, and 2%) were administrated to the LD, MD, and HD groups, respectively, as a dietary supplement for 30 consecutive days. All rabbits were included in the analysis of the growth performance, diarrhea frequency, and mortality rate. In consideration of animal ethics and welfare, only six animals from each group were sacrificed to collect additional data, despite there being 30 animals in each group. The ileal and cecal samples were collected and preserved in liquid nitrogen.

### 2.6 Growth performance

All Rex rabbits were weighed on Day 1 and Day 30 of the experiment, and the feed intake per cage was recorded daily. The average daily feed intake (ADFI) and average daily gain (ADG) were calculated. The feed conversion ratio (FCR) was expressed as ADFI/ADG.


$$\mathrm{ADFI}=\frac{\mathrm{Total}\;\mathrm{consumption}\;\mathrm{of}\;\mathrm{feed}}{\mathrm{Feeding days}\times \mathrm{rabbit}\;\mathrm{number}}$$

$$ADG=\frac{\mathrm{Final weight}-\mathrm{initial weight}}{\mathrm{Feeding days}}$$

$$FCR=\frac{\mathrm{ADFI}}{ADG}$$

### 2.7 Diarrhea and mortality percentage

The total number of diarrhea frequency of each Rex rabbit in all groups was recorded every day during the test. The number of dead Rex rabbits were recorded in each group every day during the trial period. Meanwhile the total number of dead Rex rabbits were counted. P_diarrhea percentage_ was calculated as Equation 1, while P_mortality percentage_ was calculated as Equation 2.


(1) $$p_{\mathrm{diarrhea percentage}}=\frac{\sum A}{\sum B}\times 100\%$$

Where A represents the total times of diarrhea; B represents the sum of survival days of each rabbit in the group.


(2) $$p_{\mathrm{mortality percentage}}=\frac{\sum A}{\sum B}\times 100\%$$

Where A represents the total number of dead Rex rabbits; B represents total number of Rex rabbits.

### 2.8 Histomorphology

The small intestine tissues from the CON and HD groups were fixed with 4% paraformaldehyde for 7 days. Then, they were washed under running water for 30 min, placed into a pathological embedding plastic basket for dehydration to transparency with a gradient of alcohol, and finally embedded in paraffin (Zhao L. et al., 2025). The tissues were sliced into 5 μm thick sections using a slicer (Ultra-Thin Semiautomatic Microtome, RM2235, from Leica, Germany), flattened in warm water, mounted on slides, and baked at 60°C for 2 h. Then, the sections were placed in xylene to remove the paraffin and stained with hematoxylin, eosin, or Alcian blue. The tissues were dehydrated with 85% alcohol for 5 min, 95% alcohol for 5 min, and 100% alcohol for 10 min. Finally, the tissues were placed in xylene to make them transparent and sealed with resin glue.

The slides were observed under the CX22 microscope (Leica, Germany), and the entire tissue slice was examined using the DM 1000 Leica microscopic imaging system. Image-Pro Plus 6.0 (Media Cybernetics, America) was used to count goblet cells in the Alcian blue-stained tissues and measure the length of the villi and the depth of the crypts. At least ten intact villi and corresponding crypts in the intestinal tissue were measured. Their ratio was calculated as follows:


$$R_{\left(V/C\right)}=\frac{L_{\mathrm{villi}}}{D_{\mathrm{crypt}}}$$

Where L_villi_ and D_crypts_ represent the length of the villi and the depth of the crypts, respectively.

### 2.9 DNA extraction, PCR, and sequencing of the intestinal microbiota

Total genomic DNA was extracted from the ileal and cecal content samples of the CON and HD groups using ZymoBIOMICS DNA isolation kits (ZymoBIOMICS, California, USA) according to the manufacturer’s instructions. The 16S rRNA genes in distinct regions were amplified using specific primers and barcodes. The V3-V4 hypervariable region of the 16 s rRNA gene was amplified using a PCR instrument (Applied Bio- systems^®^ Gene Amp^®^ PCR System 9700) with primers 515F (5′-GTGCCAGCMGCCGCGGTAA-3′) and 806R (5′-GGACTACNNGGGTATCTAAT-3′). All PCR mixtures contained 15 μL of the Phusion^®^ High-Fidelity PCR Master Mix (New England Biolabs), 0.2 μM of each primer, and 10 ng of target DNA. The cycling conditions consisted of an initial denaturation step at 98°C for 1 min, followed by 30 cycles at 98°C (10 s), 50°C (30 s), and 72°C (30 s), with a final 5-min extension at 72°C. An equal volume of a loading buffer (containing SYB green) was mixed with the PCR products, and electrophoresis was performed on a 2% agarose gel for DNA detection. The PCR products were then mixed in equal proportions, and the Qiagen Gel Extraction Kit (Qiagen, Germany) was used to purify the mixed PCR products. Furthermore, the DNA library was established using the TruSeq DNA PCR-Free Sample Prep Kit (Illumina, FC-121-3001/3003) according to standard protocols.

### 2.10 16S rRNA gene high-throughput sequencing

Species notes: The raw reads obtained from sequencing were filtered using the Trimmomatic software; primer sequences were identified and removed using the Cutadapt software to obtain clean reads without primer sequences. The DADA2 method in QIIME2 was used for denoising, merging paired-end sequences, and removing chimera sequences to obtain amplicon sequence variants (ASVs). The ASV feature sequences were compared with the reference sequences in the SILVA database to obtain classification information for each ASV. Using the QIIME2 software, the sequence number of each sample in the ASV abundance matrix was randomly extracted at different depths, and the sequence number extracted at each depth and the corresponding ASV number were used to draw rarefaction curves.

Alpha diversity analysis: The Dominance index, the Chao1 index, Observed_asvs, the Shannon index, the Simpson index, and Pielou_e of each group were calculated to compare the richness and evenness of the ASVs between the different samples.

Beta diversity analysis: Principal coordinate analysis (PCoA) and non-metric multidimensional scaling (NMDS) were used for visualization.

Characteristic microbiota analysis: The QIIME2 software was used to obtain composition and abundance tables for each sample at different taxonomic levels, which were presented as bar charts. To further validate the regulatory effects of MYPFG on the intestinal microbiota of Rex rabbits, we performed an LEfSe analysis to further characterize distinguishing phylotypes in the intestinal microbiota of the different groups, using a linear discriminant analysis (LDA) score of 4 as the screening criterion. Microorganisms with LDA scores greater than the set value were considered biomarkers with statistically significant differences. Multiple comparisons were made among the groups to identify characteristic genera with significant differences between them.

### 2.11 Sample preparation and UHPLC–MS of the untargeted metabolomics

The cecal content samples were individually ground with liquid nitrogen, and the homogenate was resuspended in prechilled 80% methanol using thorough vortexing. The samples were incubated on ice for 5 min and then were centrifuged at 15,000 *g*, 4°C for 20 min. Some of the supernatant was diluted to a final concentration of 53% methanol using LC–MS grade water. The samples were subsequently transferred to a fresh Eppendorf tube and were centrifuged at 15,000 *g*, 4°C for 20 min. Finally, the supernatant was injected into the LC–MS/MS system for analysis. UHPLC–MS/MS analyses were performed using a Vanquish UHPLC system (Thermo Fisher, Germany) coupled with an Orbitrap Q Exactive^™^ HF-X mass spectrometer (Thermo Fisher, Germany) at Novogene Co., Ltd. (Beijing, China). The samples were injected into a Hypesil Gold column (100 × 2.1 mm, 1.9 μm) using a 17-min linear gradient at a flow rate of 0.2 mL/min. The eluents for the positive polarity mode were eluent A (0.1% FA in Water) and eluent B (Methanol). The eluents for the negative polarity mode were eluent A (5 mM ammonium acetate, pH = 9.0) and eluent B (Methanol). The solvent gradient was set as follows: 2% B, 1.5 min; 2–85% B, 3 min; 100% B, 10 min; 100–2% B, 10.1 min; and 2% B, 12 min. The Q Exactive^™^ HF-X mass spectrometer was operated in positive/negative polarity mode with a spray voltage of 3.5 kV, a capillary temperature of 320°C, a sheath gas flow rate of 35 psi, an aux gas flow rate of 10 L/min, an S-lens RF level of 60, and an aux gas heater temperature of 350°C. The QC samples were injected into the UPLC–MS system at the beginning and end of the entire analysis process, as well as after every three test samples.

### 2.12 Metabolomics analysis

Principal component analysis (PCA) and partial least squares discriminant analysis (PLS-DA): The peaks extracted from all experimental and QC samples were analyzed using PCA. In the PCA of the QC samples in both positive and negative ion modes, the QC samples (purple) were tightly clustered and clearly separated from the experimental samples, indicating that the data were of good quality and that the instrument was stable. To obtain the biomarkers of MYPFG regulating the contents of the cecum, we used PLS-DA to analyze the differences in the metabolites between the model group and the blank group.

Volcanic map: The volcanic map screening of differential metabolites mainly involves three parameters: VIP, FC, and *p*-value. VIP refers to the Variable Importance in the Projection of the first principal component of the PLS-DA model, with the VIP value representing the contribution of metabolites to the grouping. FC refers to fold change, which is the ratio of the mean value of all biological replicate quantitative values for each metabolite in the comparison group. The threshold values were set as VIP > 1.0, FC > 1.2 or FC < 0.833, and a *p*-value of <0.05.

KEGG: KEGG was used for metabolic analysis and metabolic network research in the organisms. Pathway enrichment analysis can identify the main biochemical metabolic pathways and signal transduction pathways involved with differential metabolites. Using the hypergeometric test method, *p*-values for pathway enrichment were obtained, with a *P*_value_ of ≤0.05 set as the threshold. KEGG pathways that met this condition were defined as significantly enriched in the differential metabolites. Based on the enrichment results, we drew the bubble diagram of the enriched KEGG pathways.

### 2.13 Correlation analysis between the intestinal microbiota and metabolites

Based on the Pearson correlation coefficient, correlation analysis was conducted between the intestinal microbiota with significant differences at the genus level obtained from the 16S rDNA analysis and the metabolites with significant differences obtained from the metabolomics analysis. A heatmap was drawn to measure the degree of association between the species diversity and metabolites in the environmental samples. Another heatmap was drawn to measure the degree of association between the species diversity and metabolites in the intestinal samples. The range of values for the correlation coefficient was between −1 and 1. When the correlation coefficient was less than 0, it indicated a negative correlation. When it was greater than 0, it indicated a positive correlation.

### 2.14 Statistical analysis

A one-way analysis of variance (ANOVA) was used to analyze the experimental data with SPSS 25.0. All values were presented as mean ± SD, and a *p*-value of <0.05 was considered statistically significant.

## 3 Results

### 3.1 Growth

As shown in Table 2, the average daily gain (ADG) of the weaned Rex rabbits in the HD and MD groups significantly increased compared to the CON groups (*p* < 0.05). MYPFG significantly reduced the feed-to-meat ratio (FCR) in the weaned Rex rabbits (*p* < 0.05), indicating that MYPFG could promote growth.

**Table 2:** Effects of MYPFG on the growth performance of Rex rabbits.

Index	CON	LD	MD	HD
ADFI/(g/d)	48.08 ± 0.82	48.07 ± 1.93	48.09 ± 1.45	47.77 ± 2.60
ADG/(g/d)	5.17 ± 0.24c	7.00 ± 1.00bc	9.33 ± 0.33b	15.33 ± 2.31a
FCR	9.31 ± 0.27a	6.91 ± 0.45b	5.26 ± 0.24c	3.15 ± 0.33d

Data are expressed as mean ± SD (*n* = 6). In the same row, values with no letters or the same letter superscripts indicate no significant difference (*p* > 0.05), while values with different small letters indicate a significant difference (*p* < 0.05).

### 3.2 Diarrhea frequency and mortality rate

As shown in Table 3, MYPFG significantly reduced the frequency of diarrhea in the weaned Rex rabbits compared to the CON group (*p* < 0.05). The HD group exhibited the most obvious effect. Compared to the CON group, the mortality rate in the three test groups was also significantly reduced (*p* < 0.05). The HD group exhibited the most significant effect in reducing both diarrhea frequency and mortality in the weaned Rex rabbits, indicating that MYPFG can alleviate the effects of diarrhea on the growth of weaned Rex rabbits and help maintain intestinal health.

**Table 3:** Effects of MYPFG on diarrhea frequency and mortality percentage in Rex rabbits.

Index	CON	LD	MD	HD
Diarrhea percentage (%)	7.67 ± 0.25a	6.22 ± 0.25b	4.33 ± 0.25c	2.33 ± 0.25d
Mortality percentage (%)	86.67 ± 7.46a	36.67 ± 7.46b	23.33 ± 9.13c	13.34 ± 7.46c

Data are expressed as mean ± SD (*n* = 6). In the same row, values with no letters or the same letter superscripts indicate no significant difference (*p* > 0.05), while values with different small letters indicate a significant difference (*p* < 0.05).

According to the clinical effects observed in the different dose groups, the diarrhea and mortality rates of the HD group were significantly lower than those of the other groups. The growth performance of the HD group was also significantly better than the other groups. Subsequent experiments used the HD group as the YPF group to explore the inhibitory mechanism of MYPFG on post-weaning diarrhea in Rex rabbits.

Based on the clinical effects observed in the different dose groups, the diarrhea and mortality rates in the HD group were significantly lower than those in the other groups. The growth performance of the HD group was also significantly better than that of the other groups. Subsequent experiments used the HD group as the YPF group to explore the inhibitory mechanism of MYPFG on post-weaning diarrhea in Rex rabbits.

### 3.3 Effects of MYPFG on the intestinal mucosal structure

Histopathological examination was used to evaluate the integrity of the intestinal tissue. At the end of the experiment, tissue samples from the duodenum, jejunum, and ileum were collected and sectioned for HE staining. The results of the HE staining assay demonstrated that, in the CON group, part of the intestinal villi was shed, the intestinal villi were shortened, the lamina propria was wide, and the connective tissue in the lamina propria was loosened. However, the results of the YPF group showed that the intestinal structure was intact, the stage was clear, and the intestinal villi were neatly arranged in all groups. The tissue structure of the samples in all groups was normal, with no obvious histopathological damage found. Occasionally, the mucosal epithelium showed cell abscission and necrosis.

As shown in Table 4, MYFPG increased the length of the villi, the depth of the crypt, and their ratio R(V/C). Moreover, the depth of the crypt and R(V/C) were significantly increased in the jejunum in the YPF group, suggesting that the maturity of the jejunum in the YPF group was better. Overall, MYPFG provided better protection for the intestinal mucosa, especially in the jejunum.

**Table 4:** Effects of MYPFG on R_(V/C)_ in the intestines of the weaned Rex rabbits.

Group	Item	Duodenum	Jejunum	Ileum
CON	L_villi_ (μm)	828.71 ± 156.19	565.98 ± 80.64	432.68 ± 111.49
YPF		832.75 ± 120.16	590.90 ± 86.89	486.33 ± 145.57
CON	D_crypt_ (μm)	109.36 ± 35.39	112.27 ± 20.48	99.31 ± 20.60
YPF		110.44 ± 24.86	100.91 ± 14.58*	100.87 ± 24.26
CON	R _(V/C)_	7.98 ± 1.90	4.85 ± 1.32	4.64 ± 1.78
YPF		7.85 ± 1.78	5.98 ± 1.21*	4.87 ± 1.30

Data are expressed as mean ± SD (*n* = 6).

The asterisk (*) indicates that there is a significant difference between the YPF group and the CON group (*p* < 0.05).

### 3.4 Effects of MYPFG on the morphology of the goblet cells in the intestines

The main function of goblet cells is to synthesize and secrete mucin, forming a mucosal barrier to protect epithelial cells. The number of goblet cells indicates the protective effect of the intestinal mucus barrier. The number of goblet cells in the duodenum, jejunum, and ileum of the YPF group increased (Table 5). In general, the number of goblet cells increased insignificantly, possibly because MYPFG had a minimal effect on the intestinal mucus barrier.

**Table 5:** The number of goblet cells in the duodenum, jejunum, and ileum in the same visual field.

Group	Duodenum	Jejunum	Ileum
CON	30.17 ± 3.90	36.50 ± 6.50	66.58 ± 18.83
YPF	36.00 ± 6.94	42.08 ± 4.08	71.92 ± 16.94

### 3.5 Effect of MYPFG on the intestinal microbiota

**Figure 1:**
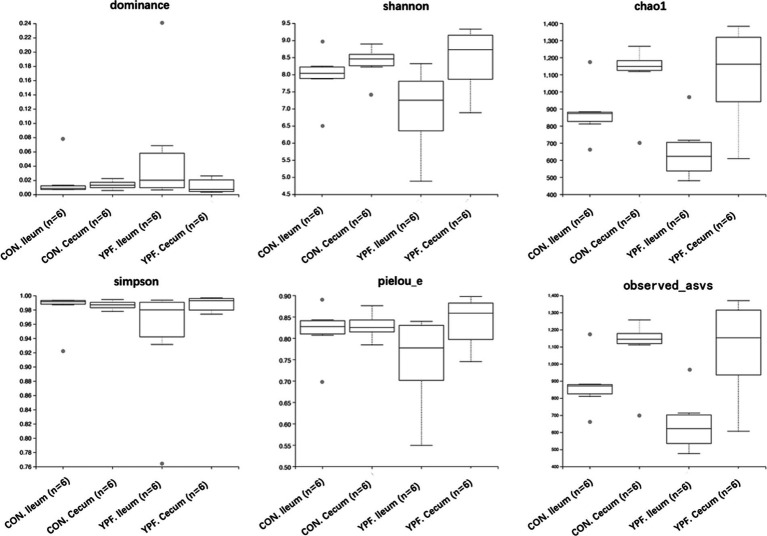
Dominance, shannon, chaol, simpson, pielou e, observed asvs.

**Figure 2:**
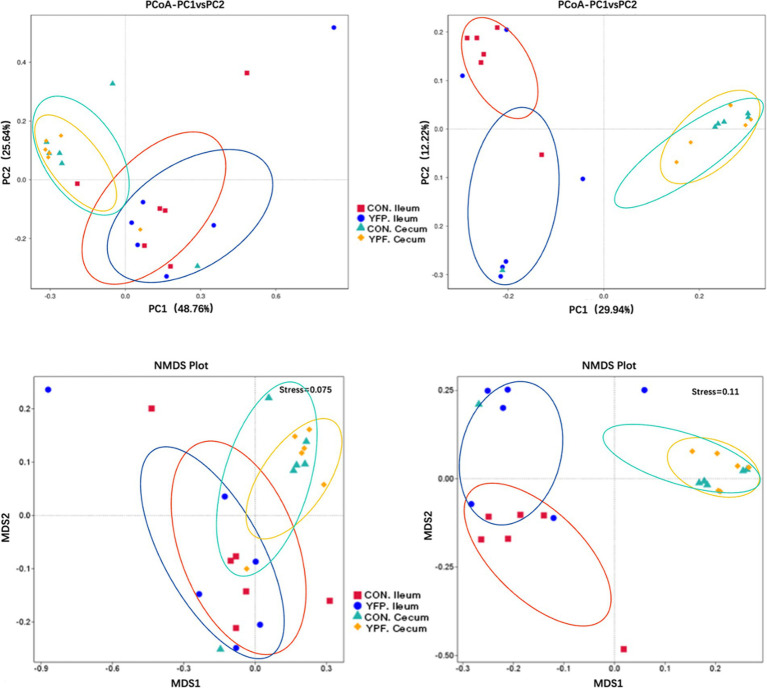
PCoA and NMDS analysis of each group. Left: Based on Weighted Unifrac distance. Right: Based on Unweighted Unifrac distance. Weighted Unifrac distance is more sensitive to species with higher abundance, while Uweighted Unifrac distance is more sensitive to rare species.

In the ileal microbiota, the CON group had 3,565 ASVs, with 2,766 unique ASVs, while the YPF group had 2,866 ASVs, with 2,067 unique ASVs. The two ileum groups shared a total of 5,632 ASVs. In the cecal microbiota, the CON group had 3,405 ASVs, with 1,616 unique ASVs, while the YPF group had 3,190 ASVs, with 1,401 unique ASVs. The two cecum groups shared a total of 4,806 ASVs. The results showed that MYPFG had a powerful effect on changing the intestinal microbiota, especially the ileal microbiota. This finding indicated that the MYPFG treatment significantly altered the species composition and diversity of the intestinal microbiota in Rex rabbits. As shown in Figure 1, alpha diversity analysis of the microbial communities was performed to compare species richness (Dominance index, Chao1 index, and Observed_asvs) and diversity (Shannon index, Simpson index, and Pielou_e) among the two ileum groups and the two cecum groups. MYPFG effectively altered the species richness and diversity of the microbiota in Rex rabbits. In PCoA and NMDS analyses (Figure 2), the closer the distance between two points on the coordinate axis, the more similar their community composition is in the respective dimensions. Based on the weighted UniFrac distance, the PCoA analysis showed that the contribution rate of the horizontal axis (PCoA1) was 48.76% and the contribution rate of the vertical axis (PCoA2) was 25.64%. Based on the unweighted UniFrac distance, the PCoA analysis showed that the contribution rate of the horizontal axis (PCoA1) was 29.94% and the contribution rate of the vertical axis (PCoA2) was 12.22%. The ileum samples were completely separated from the cecum samples. The CON samples partially overlapped with the MYPFG samples between the ileum groups. Similar results were also observed between the cecum groups. The differences in the composition of the intestinal microbiota could be identified in the PCoA analysis and NMDS analysis. The results of the NMDS analysis were consistent with those of the PCoA analysis, indicating that MYPFG influenced the body functions by altering the intestinal microbiota.

**Figure 3:**
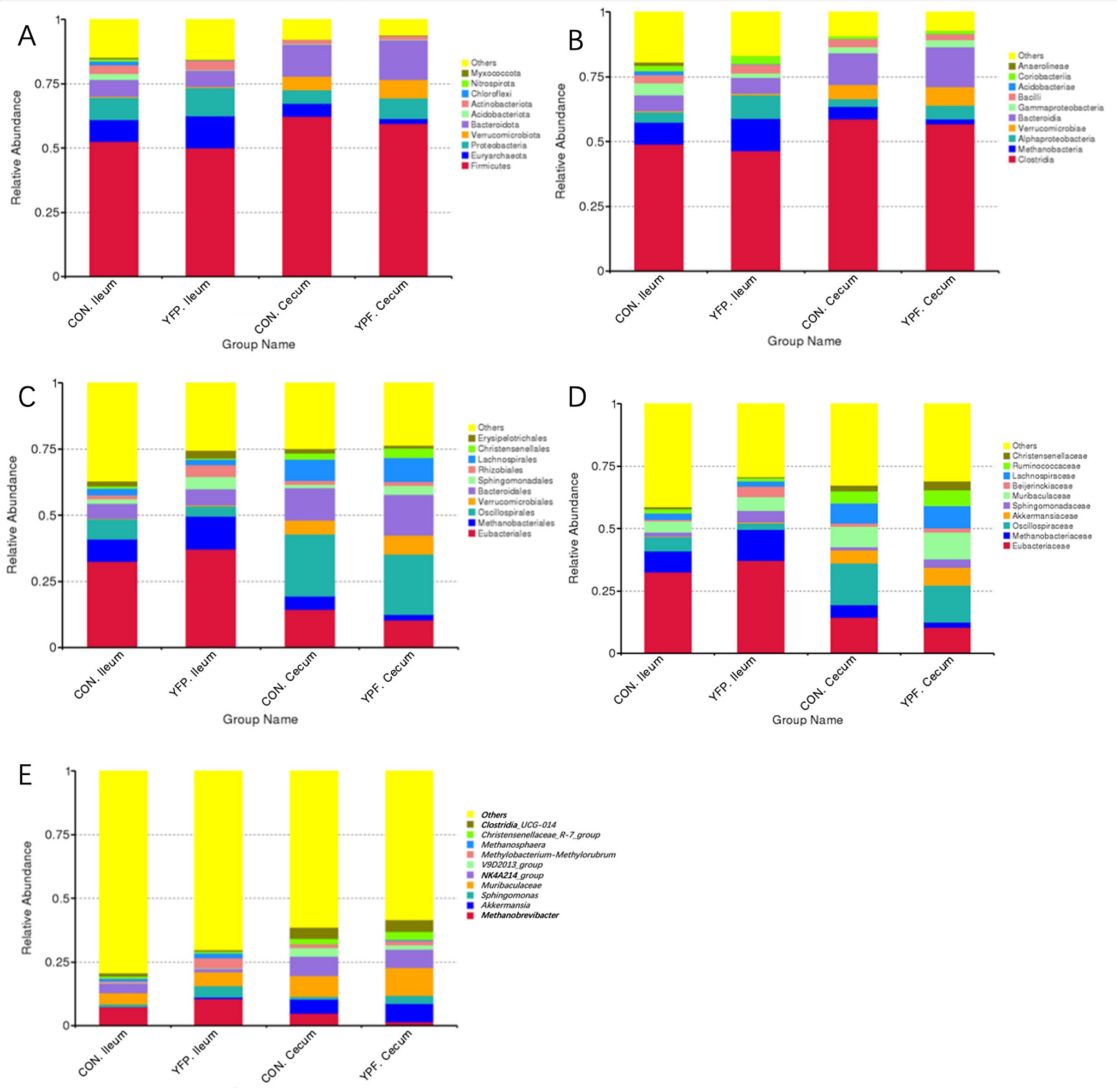
Effect ofYPF on intestinal microbiota abundance and specific taxa. Differences in the abundance of bacteria in each group at the **(A)** phylum, **(B)** class, **(C)** order, **(D)** family, **(E)** genus level.

To investigate the effect of MYPFG on the intestinal microbiota of Rex rabbits, we selected the top 10 dominant phyla and genera with a relative abundance of more than 1% and used bar charts to represent their abundance. Comparing the structural changes of the intestinal microbiota in each group based on the ASVs, Myxococcota, Nitrospirota, Chloroflexi, Actinobacteriota, Acidobariota, Bacteroidota, Verrucomicrobiota, Proteobacteria, Euryarchaeota, and Firmicutes exhibited higher abundance at the phylum level (Figure 3A). The top 10 classes with the highest abundance at the class level were Anaerolineae, Coriobacterriia, Acidobacteriae, Bacilli, Grammaproteobacteria, Bacteroidia, Verrucomicrobiae, Alphaproteobactaria, Methanobacteria, and Clostridia (Figure 3B). The orders with relatively high abundance at the order level were Ersipelotrichales, Christensenellales, Laphnospirales, Rhizobiales, Sphingomonadales, Bacteroidales, Verrucomicrobiales, Oscillospirales, Methanobacteriales, and Eubacteriales (Figure 3C). Similarly, we conducted an analysis at the family level. The 10 families with relatively high abundance were Christensenellaceae, Ruminococcaceae, Lachnospiraceae, Beijerinkiaceae, Muribaculaceae, Sphingomonadaceae, Akkermansiaceae, Oscillospiraceae, Methanobacteriaceae, and Eubacteriaceae (Figure 3D). At the genus level, the higher abundance of *Clostridia-UGG-014*, *Christensenellaceae-R-7-group*, *Methanosphaera*, *Methylobacterium-Methylorubrum*, *V9D2013-group*, *NK4A214-group*, *Muribaculaceae*, *Sprhingomonas*, *Akkermansia*, and *Methanobrevibacter* indicated that these genera played an important role in each group (Figure 3E).

**Figure 4:**
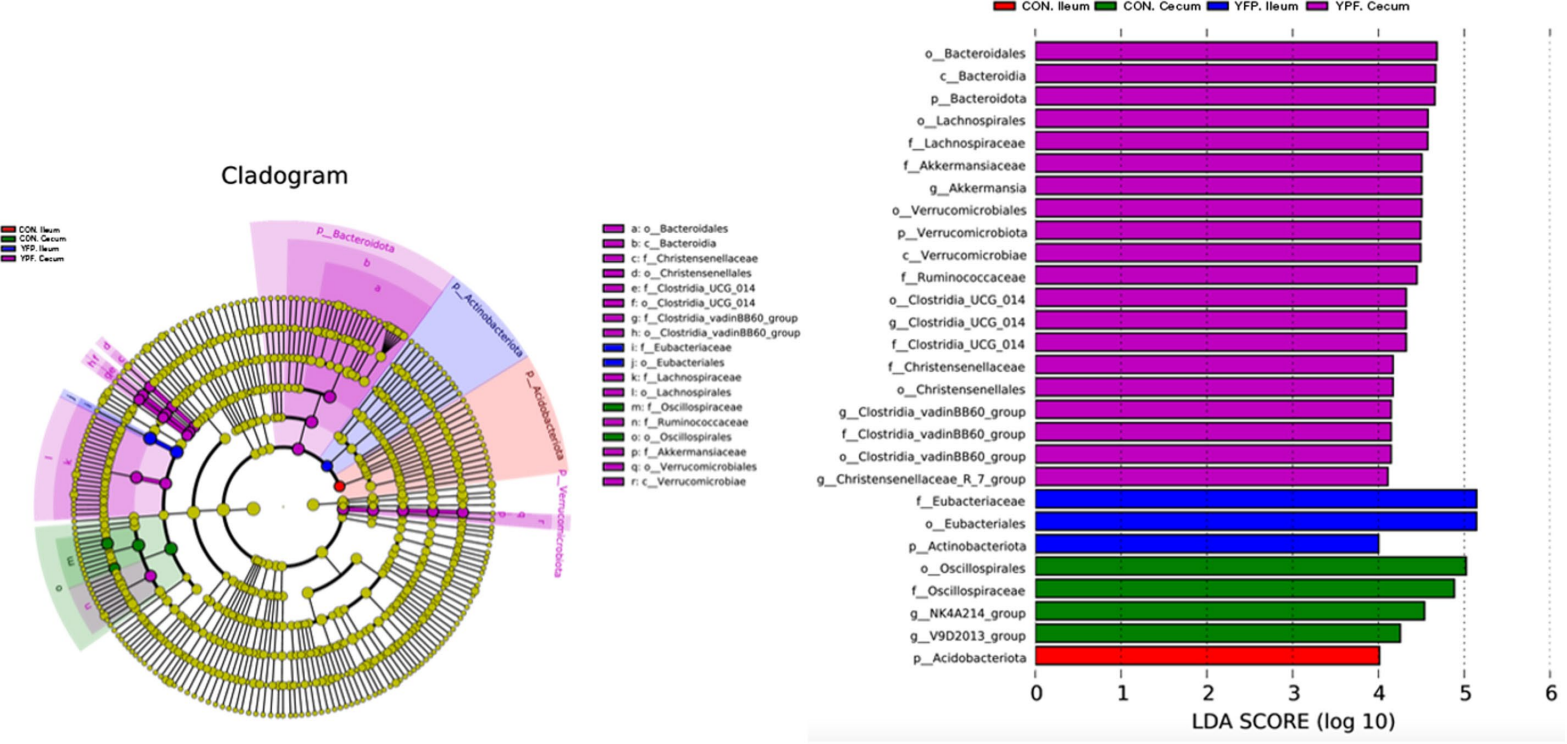
Expression of important microbiota in each group.

**Figure 5:**
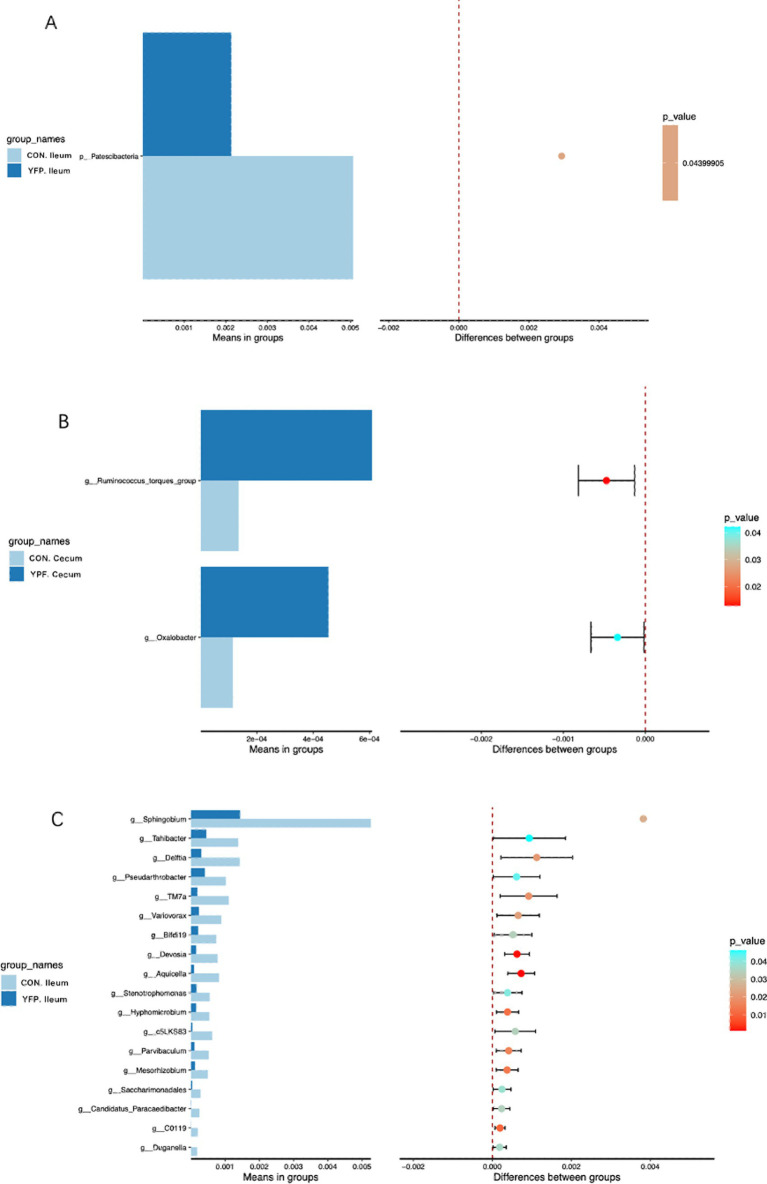
The abundances of species with significant differences between groups. **(A)**: phylum level; **(B,C)**: genus level.

Figure 4 shows the microbiota with significant differences in abundance. The length of the histogram represents the impact of the significantly different species. Bacteroidales, Bacteroidia, Bacteroidota, Lachnospirales, Lachnospirales, Akkermansiaceae, Akkermansia, Verrucomicrobiales, Verrucomicrobiae, Clostridia, Christensenellaceae, Christensenellales, Clostridia, and Christensenellaceae exhibited higher expression in the YPF cecum group, while Oscillosprirales, Oscillosprirales, NK4A214, and V9D2013 exhibited higher expression in the CON cecum group. Acidobacteriota exhibited higher expression in the CON ileum group, while Eubacteriaceae, Eubaceriales, and Actinobacteriota exhibited higher expression in the YPF ileum group. The results of the study showed that the structure of the cecal microbiota underwent significant changes after the MYFPG treatment. The results also showed that the intestinal microbiota plays an important role in the prevention and treatment of post-weaning diarrhea. The results of the species with significant differences in abundance between the YPF group and the CON group are shown in Figure 5. The abundance of Patencibacteria in the YPF ileum group was significantly lower than that in the CON ileum group. The abundance of *Sphingobium* in the YPF ileum group was also significantly lower than that in the CON ileum group. The abundance of *Oxalobacter* and *Ruminococcus* in the YPF cecum group was significantly higher than that in the CON cecum group.

### 3.6 Effect of MYPFG on the metabolomics of the cecal contents

**Figure 6:**
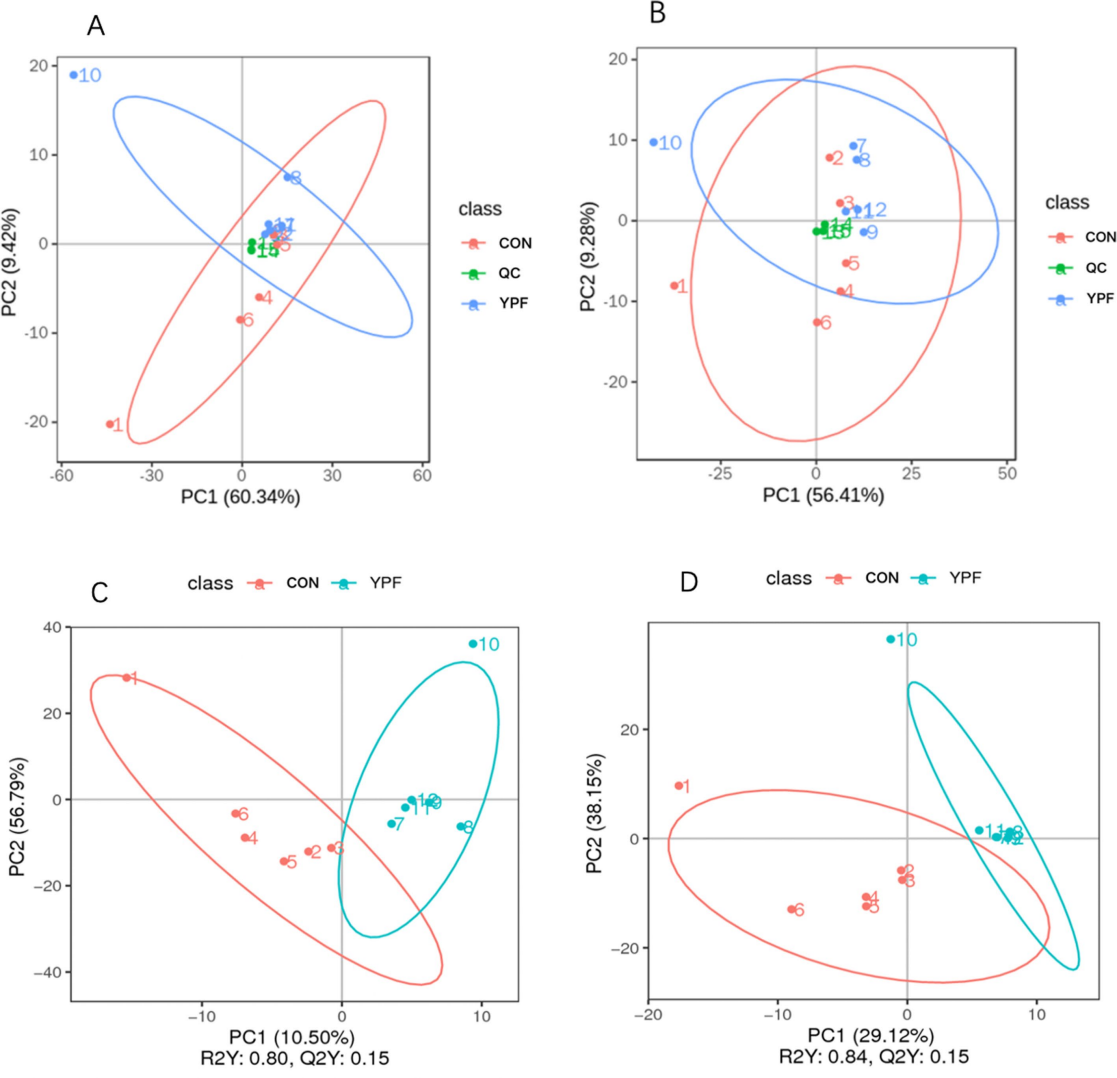
Effect of MYPFG on metabolites. **(A)** PCA in positive ion mode. **(B)** PCA in negative ion mode. **(C)** PLS-DA in positive ion mode. **(D)** PIS-DA in negative ion mode.

**Figure 7:**
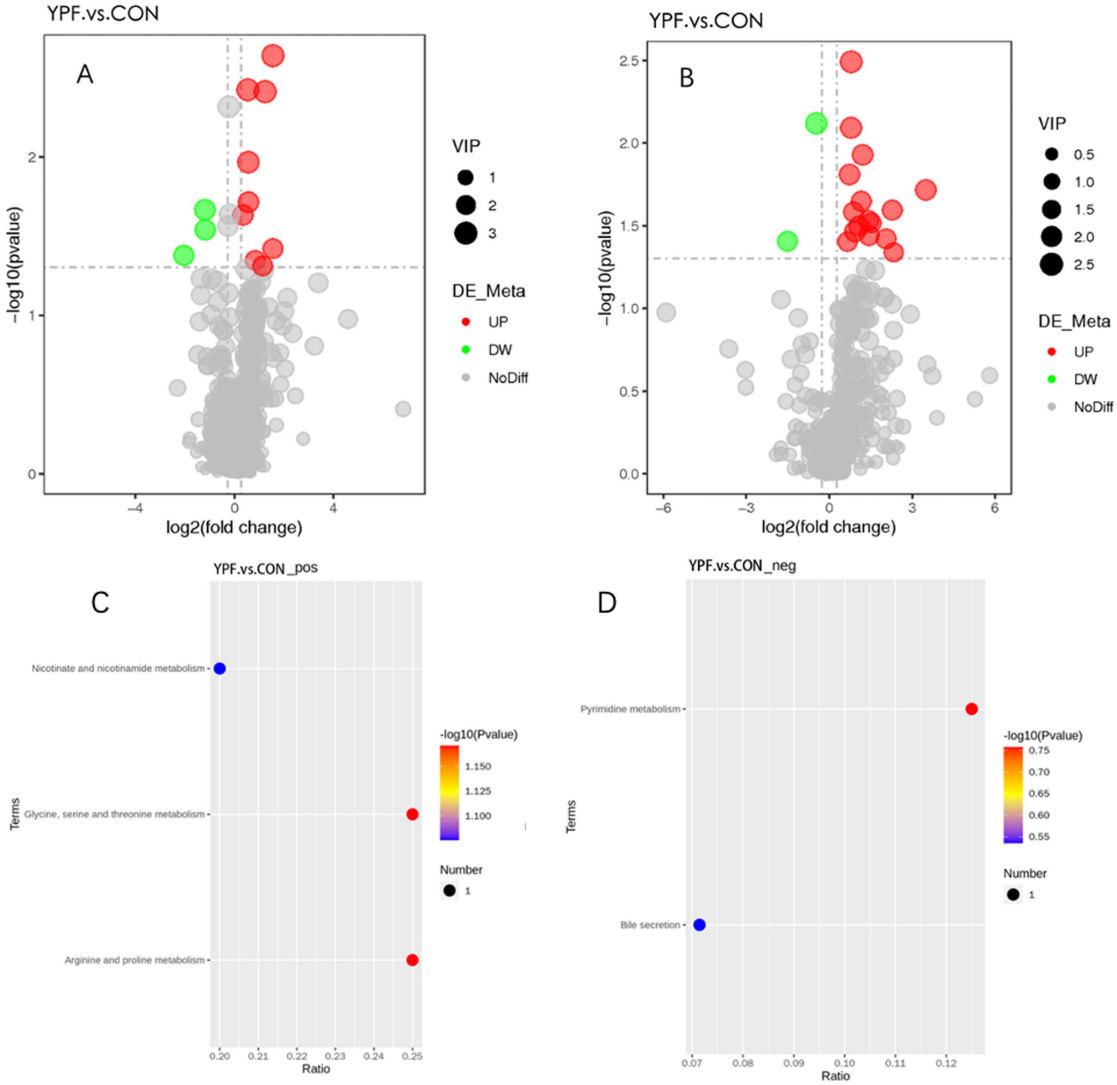
Volcano plot of the differential metabolites between the YPF group and the CON group under positive **(A)** and negative **(B)** ion mode. Enrichment analysis of differential metabolite pathways between the YFP group and the CON group under positive **(C)** and negative **(D)** ion mode.

In the PCA (Figures 6A,B), the horizontal axis PC1 and vertical axis PC2 in the figure represent the scores of the first- and second-ranked principal components, respectively. Different colored scatter points represent samples from different experimental groups, and the ellipse indicates the 95% confidence interval. In the positive ion mode, the PCA analysis showed that the contribution rate of the horizontal axis PC1 was 60.34% and the contribution rate of the vertical axis PC2 was 9.42%. In the negative ion mode, the PCA analysis showed that the contribution rate of the horizontal axis PC1 was 56.41% and the contribution rate of the vertical axis PC2 was 9.28%. In the positive ion PLS-DA (*R*^2^ = 0.80, *Q*^2^ = 0.15), most of the samples were separated, indicating differences in the metabolite levels between the two groups (Figure 6C). In the negative ion PLS-DA (*R*^2^ = 0.84, *Q*^2^ = 0.15), most of the samples were also separated (Figure 6D). The volcanic map directly displays the overall distribution of differential metabolites (Figures 7A,B). In the positive ion mode, nine differential metabolites were upregulated and three were downregulated. The screened differential metabolites are shown in Table 6. In the negative ion mode, 16 differential metabolites were upregulated and two were downregulated. The screened differential metabolites are shown in Table 7. In the KEGG pathway enrichment (Figures 7C,D), the horizontal axis in the figure represents the number of differential metabolites in the corresponding metabolic pathway and the vertical axis represents the total number of metabolites identified in the pathway. The results showed that nicotinate and nicotinamide metabolism, glycine, serine, and threonine metabolism, and arginine and proline metabolism were enriched in the positive ion mode. Pyrimidine metabolism and bile metabolism were enriched in the negative ion mode.

**Table 6:** Identified potential biomarkers in the cecal contents in the positive ion mode.

No.	Metabolite	FC	VIP	Up/Down
1	4-oxo-4-[(1-phenylethyl)amino]but-2-enoic acid	2.93	3.16	Up
2	N-Tetradecanamide	1.45	3.11	Up
3	Linolelaidic acid (C18:2N6T)	2.36	3.14	Up
4	(2E,4E)-N-(2-methylpropyl)dodeca-2,4-dienamide	1.48	2.97	Up
5	(2E,4E)-N-(2-methylpropyl)deca-2,4-dienamide	1.48	2.69	Up
6	Creatine	0.44	2.67	Down
7	N-(9-oxodecyl)acetamide	1.27	2.62	Up
8	5α-Pregnan-3,20-dione	0.44	2.56	Down
9	6-(3-hydroxybutan-2-yl)-5-(hydroxymethyl)-4-methoxy-2H-pyran-2-one	2.94	2.45	Up
10	Guanidineacetic acid	0.24	2.51	Down
11	Nicotinic acid	1.79	2.40	Up
12	Linoleoyl ethanolamide	2.21	2.34	Up

**Table 7:** Identified potential biomarkers in the cecal contents in the negative ion mode.

No.	Metabolite	FC	VIP	Up/Down
1	(±)18-HEPE	1.73	2.51	Up
2	Deoxycholic acid	0.72	2.36	Down
3	Hematoxylin	1.73	2.31	Up
4	1-[(1R,2S,3R,5R)-5-Cyclohexyl-2,3-dihydroxycyclopentyl]-3-ethylurea	2.31	2.21	Up
5	Ferulic acid	1.66	2.17	Up
6	4-(morpholinosulfonyl)aniline	11.28	2.19	Up
7	Indoxylsulfuric acid	2.23	2.04	Up
8	Thymidine 5′-monophosphate	4.85	2.04	Up
9	N-{[2-(2-thienyl)-1,3-thiazol-4-yl]methyl}benzamide	1.84	2.03	Up
10	8-iso-15-keto Prostaglandin F2α	2.68	2.06	Up
11	4-Hydroxy-2-Oxoglutaric acid	2.87	1.96	Up
12	Uridine monophosphate (UMP)	2.11	1.98	Up
13	(+/−)12(13)-DiHOME	1.90	2.20	Up
14	1-acetyl-N-(6-chloro-1,3-benzothiazol-2-yl)-4-piperidinecarboxamide	2.71	2.00	Up
15	Uridine	4.16	2.03	Up
16	N′1-(3,4-dichlorobenzoyl)-2-(hydroxymethyl)benzene-1-carbohydrazide	0.35	2.01	Down
17	Hydroxyglutaric acid	1.58	1.89	Up
18	N1-(4-chlorophenyl)-2-cyano-4,4-dimethyl-3-oxopentanamide	5.03	1.84	Up

### 3.7 Correlation between the intestinal microbiota and metabolites

**Figure 8:**
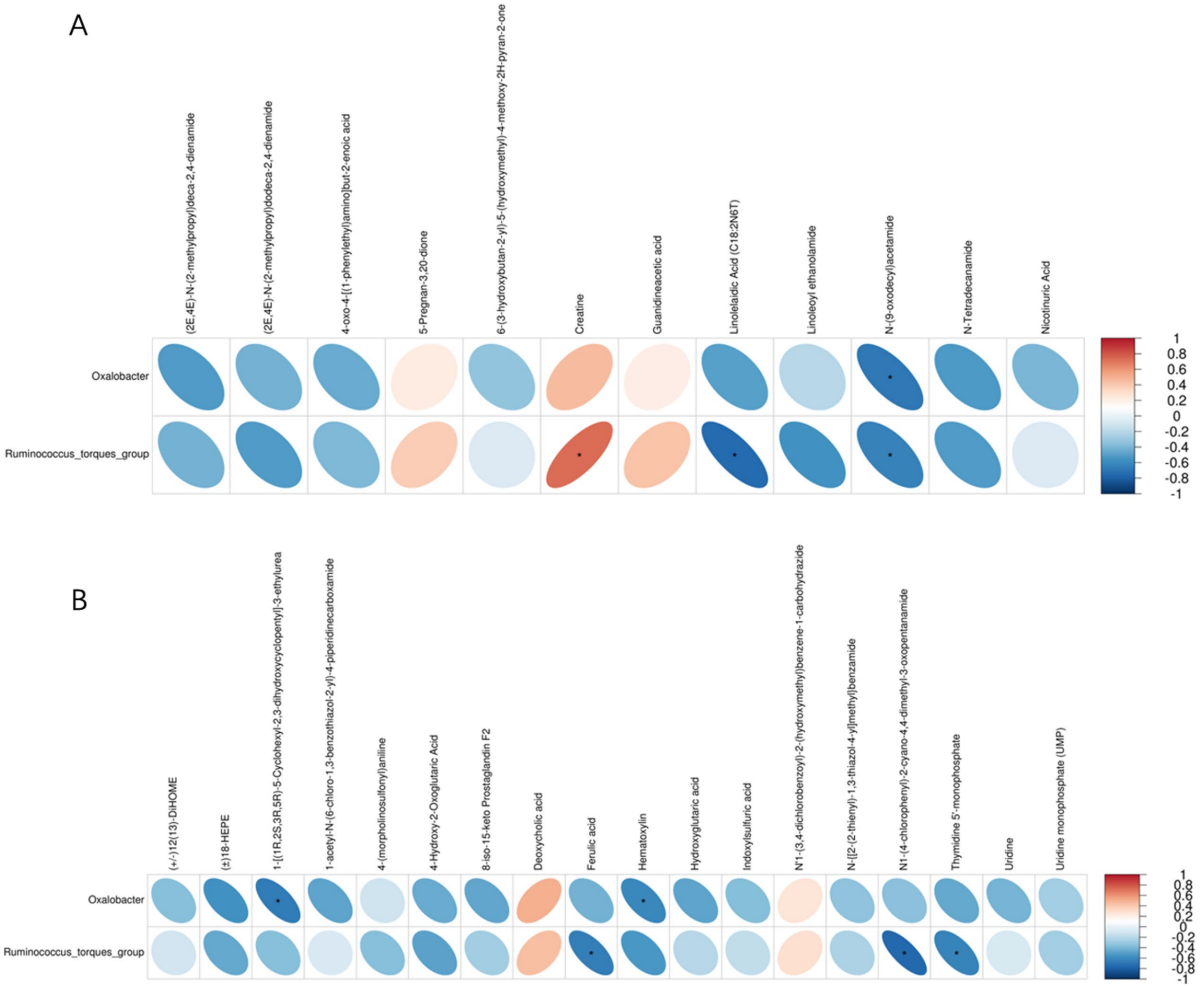
Correlations between the relative abundance of intestinal microbiota and metabolites. **(A)** Positive ion mode. **(B)** Negative ion mode. Blue indicates a negative correlation, and red indicates a positive correlation. The blank area in the figure indicates a *p* > 0.05, and the area marked by an asterisk (*) indicates *p* < 0.05.

The correlation analysis of the metabolites and intestinal microbiota showed that there was a significant correlation between *Oxalobacter* and N-(9-oxodecyl) acetamide, 1-3-ethylurea, and hematoxylin. *Ruminococcus_torques_group* was significantly correlated with creatine, linolelaidic acid, N-(9-oxodecyl) acetamide, ferulic acid, N1-(4-chlorophenyl)-2-cyano-4, 4-dimethyl-3-oxopentanamide, and thymidine 5′-monophosphate (Figure 8).

## 4 Discussion

### 4.1 MYPFG could protect the intestinal barrier

Traditional Chinese medicine (TCM) plays a crucial role in animal growth performance and intestinal barrier protection. Growth performance is the gold standard for evaluating the effectiveness of TCM additives (Li et al., 2025). Meanwhile, growth performance is also an important indicator of intestinal health and is inversely proportional to the diarrhea rate. The small intestine is an important site for nutrient absorption (Lei et al., 2022). Meanwhile, the small intestine is rich in various digestive enzymes. The longer the epithelial villi, the more epithelial cells there are. The shallower the crypt depth, the higher the rate of epithelial cell maturation (Muyang et al., 2022). The villi and crypt represent the absorptive function of the small intestine. The main effect of MYFPG on the development of the small intestine is to increase villus length, reduce crypt depth, and improve the villus-to-crypt ratio. The intestinal mucosal barrier function refers to the combined structure and function that prevent harmful substances from crossing the intestinal mucosa and entering other tissues, organs, and the bloodstream (Zhao L. et al., 2025). Barrier proteins can organize harmful substances to damage the intestines, brain, and reproductive glands (Li M. Y. et al., 2024; Zhao L. et al., 2024). The protective effect of the intestinal mucosa is closely related to goblet cells. Goblet cells can synthesize and secrete mucin needed for the intestinal mucosal barrier (Ma et al., 2020). Sijunzi decoction has been shown to be effective in improving the immunity of the small intestine and ensuring the integrity of the mucosal barrier in weaned Rex rabbits (Li et al., 2022). Another report showed that TCM could improve the growth performance of weaned Rex rabbits (Ning et al., 2018). Berberine can alleviate diarrhea and improve intestinal mucosal barrier function in piglets (Du et al., 2025). It has been shown that long-term administration of total glucosides of *Paeonia lactiflora* improves intestinal epithelial barrier damage in rats (Rui et al., 2023). Our research showed that MYFPG can increase the number of goblet cells, increasing the protective effect of the intestinal mucosal barrier.

### 4.2 MYPFG could alleviate diarrhea by regulating the intestinal microbiota

TCM is advantageous for treating diarrhea and regulating the intestinal microbiota. Our study showed that MYPFG could regulate the intestinal microbiota in Rex rabbits to decrease post-weaning diarrhea. Similarly, Lingguizhugan decoction could regulate the intestinal microbiota to treat diarrhea by promoting the growth of Muribaculaceae and *Dubosiella*, while inhibiting the growth of Christensenellaceae, Christensenellaceae_R_7_group, and UCG_005 (Huang et al., 2024). Christensenellaceae and Christensenellaceae_R_7_group also played an important role in our study, as they were key intestinal microbiota associated with diarrhea. It was reported that the Yangyin-ningshen formula could also decrease the abundance of Actinobiota while increasing the abundance of Bacteriodia to treat diarrhea (Lei et al., 2024). However, Actinobiota and Bacteriodia did not appear in the MYPFG regulation intestinal microbiota. Another report showed that the TCM Shenling Baizhu decoction could regulate *Monoglobus*, *Dubosiella*, and *Akkermansia* to treat diarrhea (Shao et al., 2024). The abundance of *Akkermansia* was also changed by MYPFG in our intestinal microbiota results. Rice water-fried Atractylodis Rhizoma could treat diarrhea by significantly enriching beneficial bacteria, such as *Lactobacillus*, and decreasing the abundance of potentially pathogenic fungi, such as *Aspergillus* (Chunping et al., 2023). However, neither *Lactobacillus* nor *Aspergillus* appeared in our research, which may be related to the use of different experimental animals. We found that, after being given MYPFG, the abundance of *Ruminococcus* significantly increased, leading to an increase in the secretion of carbohydrate-degrading enzymes and an increase in their ability to absorb carbohydrates from solid feed. The result is consistent with those of a study conducted by Zhao M. et al. (2024) which showed a significant increase in *Ruminococcus* in the intestinal microbiota of weaned rabbits. It was reported that adding carbohydrate-degrading enzymes to the diet can improve the intestinal health of weaned piglets (Ribeiro et al., 2024), which is consistent with our study. At the same time, the abundance of *Oxalobacter* significantly improved under the influence of MYPFG, which greatly enhanced the ability of otter rabbits to absorb oxalic acid from feed in their intestines, eliminating the phenomenon of oxalic acid accumulation in the intestines due to poor absorption (Pegah and John, 2022). The abundance of *Ruminococcus_torques_group* and *Oxalobacter* was significantly higher in the YPF cecum groups than in the CON cecum group at the genus level. *Ruminococcus* is associated with intestinal diseases (inflammatory bowel disease, irritable bowel syndrome, Crohn, etc.) (He C. et al., 2019; Buffet-Bataillon et al., 2024), immune diseases (allergy, eczema, asthma, etc.) (Ji et al., 2022), and nervous system diseases (autism, depression, etc.) (Morioka et al., 2022; Yan et al., 2022). It has been confirmed that *Ruminococcus* can repair intestinal mucosal barrier damage (Xia et al., 2022) and that *Oxalobacter* can degrade oxalic acid and prevent the formation of oxalate (Li et al., 2015). The colonization of *Oxalobacter* can normalize oxalic acid excretion (Canales and Hatch, 2017). Other studies have shown that *Oxalobacter* could help intestinal epithelial cells transfer oxalic acid (Donna et al., 2023). Oxalic acid can reduce the bioavailability of mineral elements, and it is easy to form calcium oxalate with calcium ions in animals, leading to kidney stones. These findings indicate that *Ruminococcus* and *Oxalobacter* play important roles in intestinal microorganisms. MYPFG can alleviate diarrhea symptoms in several ways by regulating *Ruminococcus* and *Oxalobacter*. There have been many reports on changes in the intestinal microbiota of other weaned animals after treatment with TCM. Scholars have shown that dietary *Astragalus* can regulate the intestinal immunity and intestinal microbiota of weaned piglets, and it can improve the growth performance and intestinal health of weaned piglets (Che et al., 2024). Recent studies have shown a correlation between piglet diarrhea and the intestinal microbiota, with Bacteroidaceae and Caudoviricetes being the main differential organisms strongly correlated with host status (Xie F. et al., 2024). Licorice extract could improve growth performance, enhance antioxidant capacity, and alter the abundance of the intestinal microbiota *Rikenellaceae* in weaned piglets (Zhu et al., 2024). Dietary Qi-Weng-Huangbo powder reduced diarrhea rates, improved growth performance, and enhanced immune function in weaned piglets. These improvements were potentially supported by changes in the ileum and colonic morphology and the modulation of the colonic microbial profiles (Chuanpi et al., 2023). Dietary berberine and ellagic acid supplementation could improve growth performance and reduce intestinal damage by regulating the structural function of the intestinal microbiota in weaned piglets. This regulation altered the composition of the microbiota, including Firmicutes, *Bacteroidetes*, *Lactobacillus*, *Phascolarctobacterium*, and *Parabacteroides* (Wenxia et al., 2023). Dietary berberine supplementation improves growth performance and alleviates intestinal injury in weaned piglets by modulating the ileal microbiota and metabolites (Cui et al., 2023).

### 4.3 MYPFG could alleviate diarrhea by altering the metabolites of cecal contents

Analyzing the mechanism of action of TCM through metabolomics has become a hot research topic in recent years. Some scholars have found that *Coptis chinensis* could significantly downregulate 76 metabolites and upregulate 31 metabolites to treat diarrhea (Ting et al., 2022). Another TCM, Alfalfa polysaccharides, has been shown to alleviate diarrhea by altering metabolites in calves (Zhao J. et al., 2025). It was reported that metabolomics revealed the therapeutic effect of Pueraria polysaccharides on calf diarrhea (Liuhong et al., 2023). In our study, we found 30 metabolites that were significantly different between the CON group and the YPF group based on the screening conditions. The enrichment analysis of the metabolites revealed that glycine, serine, and threonine metabolism; arginine and proline metabolism; and nicotinate and nicotinamide metabolism differed between the groups. Some studies have shown that adding glycine, serine, and threonine to diets can increase the growth performance of animals, which may be due to changes in the metabolic pathways of glycine, serine, and threonine (Ospina-Rojas et al., 2018; Hilliar et al., 2019). These metabolic pathways are closely related to diarrhea and intestinal injury in Rex rabbits. Meanwhile, it has also been shown that glycine, serine, and threonine metabolism enhances the elimination of pathogenic bacteria in the body (Chen et al., 2019). MYPFG can increase the resistance and growth performance of weaned Rex rabbits through glycine, serine, and threonine metabolism. Arginine and proline metabolism can serve as markers for functional gastrointestinal diseases, such as functional dyspepsia and irritable bowel syndrome (Karpe et al., 2022). It was also found that arginine and proline metabolism were changed in the liver injury model (Guo Chang et al., 2022). The scholars also found that animals with defects in arginine and proline metabolism would suffer from malnutrition (Abdullah et al., 2017). These findings also confirm that arginine and proline metabolism is closely related to diarrhea. Anti-inflammatory-related potential biomarkers are associated with nicotinic acid and nicotinamide metabolism (Ma et al., 2016). Another study suggested that amino acid metabolism is also related to animal stress (He J. et al., 2019). Studies on nicotinic acid and nicotinamide metabolism have shown that diarrhea is not only related to weaning but also to animal stress and inflammation. Our research showed that these amino acid metabolic pathways can be used as markers for diarrhea and that MYPFG can also treat diarrhea through multiple targets.

### 4.4 Correlation analysis between the intestinal microbiota and metabolites

Integrated metabolomics and intestinal microbiota analysis can provide us with a deeper understanding of the mechanism of MYPFG in treating diarrhea. Recently, some scholars have reported exploring the mechanism of TCM by integrating metabolomics and intestinal microbiota analysis. It was reported that integrating metabolomics and intestinal microbiota analysis revealed the prevention mechanism of TCM (Gushudan) in rats with kidney-yang deficiency syndrome (Xin et al., 2024). Another report showed that a new strategy for studying the mechanism of TCM, by integrating metabolomics and intestinal microbiota analysis together, was developed and proposed, with platycodin D as an example (Yuan-han et al., 2023). Scholars have explored the effects of Astragali Radix against cisplatin-induced liver injury using 16S rRNA gene sequencing and LC/MS-based metabolomics (Ling et al., 2022). Our study combined the cecal microbiota and the metabolomics of the cecal contents. In the correlation analysis between the cecal microbiota and cecal contents metabolites, the correlations between *Oxalobacter* and N-(9-oxodecyl) acetamide, 1-3-ethylurea, and hematoxylin, as well as the correlations between *Ruminococcus* and creatine, linolelaidic acid, N-(9-oxodecyl) acetamide, ferulic acid, N1-(4-chlorophenyl)-2-cyano-4, 4-dimethyl-3-oxopentanamide, and thymidine 5′-monophosphate, indicated their important roles in the treatment of diarrhea. MYPFG exerts an anti-diarrhea effect by regulating the relative abundance of *Ruminococcus* and *Oxalobacter* and by participating in the metabolism of glycine, serine, and threonine; arginine and proline; and nicotinate and nicotinamide. Scholars have confirmed that *Ruminococcus* can repair intestinal damage (Jiang et al., 2025). It has been reported that the abundance of *Oxalobacter* affects the absorption of oxalic acid (Lama et al., 2021). Glycine plays an important role in regulating the morphology, barrier function, and intestinal microbiota of ducks (Yaqi et al., 2023). Serine alleviates colitis by regulating intestinal *α* 1,2-fucosylation (Yao et al., 2024). Threonine could help mice excrete harmful metal ions from their intestines (Yongbin et al., 2022). Supplementing arginine in the diet could improve the growth performance of piglets through the intestinal microbiota (Liu C. et al., 2024). Proline promotes the activation of lymphoid tissue inducer cells to maintain intestinal homeostasis (Di et al., 2023). Nicotinate and nicotinamide could regulate the intestinal microbiota of rats, reduce intestinal barrier permeability, and avoid inflammatory reactions (Sai et al., 2025). All of the above-discussed similar studies indicate that MYPFG affects intestinal health from different directions, such as the abundance of intestinal microbiota and amino acid metabolism pathways.

This study investigated the protective effect of MYPFG on the intestinal tract of weaned rabbits using intestinal microbiota and metabolomics methods and explored the specific pathways through which the intestinal microbiota and metabolomics protect the intestinal tract of rabbits. In the future, we will further investigate the mechanism of action of the key active ingredients in MYPFG and conduct large-scale clinical trials to verify its effects. We will also explore the potential application of MYPFG in treating diarrhea in other animals or humans. With an increasing number of TCM treatments being able to elucidate the mechanism of treating diarrhea using modern medical methods, we believe this study has enormous value and potential for the clinical translation of TCM.

## 5 Conclusion

MYPFG could decrease post-weaning diarrhea by regulating the intestinal microbiota and metabolites. We identified 30 differentially expressed metabolites as potential biomarkers of PWD, including nicotinic acid and creatine. MYPFG regulated the abundance of Patescibacteria, *Sphingobium*, *Oxalobacter,* and *Ruminococcus* and participated in the activities of three metabolic pathways, thereby alleviating the symptoms of PWD through multiple targets and mechanisms. The results further characterized the pharmacological mechanism of MYPFG against PWD.

## Data Availability

The datasets presented in this study can be found in online repositories. The names of the repository/repositories and accession number(s) can be found at: https://www.ncbi.nlm.nih.gov/, PRJNA1084245.
